# Increase in serum DKK1 levels attenuates the anabolic response to romosozumab in postmenopausal osteoporosis

**DOI:** 10.1093/jbmr/zjaf110

**Published:** 2025-08-19

**Authors:** Giovanni Adami, Filippo Montanari, Angelo Fassio, Francesco Pollastri, Anna Piccinelli, Camilla Benini, Emma Pasetto, Mattia Tugnolli, Davide Gatti, Maurizio Rossini, Ombretta Viapiana

**Affiliations:** Rheumatology Unit, University of Verona, 37134 Verona, Italy; Rheumatology Unit, University of Verona, 37134 Verona, Italy; Rheumatology Unit, University of Verona, 37134 Verona, Italy; Rheumatology Unit, University of Verona, 37134 Verona, Italy; Rheumatology Unit, University of Verona, 37134 Verona, Italy; Rheumatology Unit, University of Verona, 37134 Verona, Italy; Rheumatology Unit, University of Verona, 37134 Verona, Italy; Rheumatology Unit, University of Verona, 37134 Verona, Italy; Rheumatology Unit, University of Verona, 37134 Verona, Italy; Rheumatology Unit, University of Verona, 37134 Verona, Italy; Rheumatology Unit, University of Verona, 37134 Verona, Italy

**Keywords:** romosozumab, Wnt system, Dkk1, sclerostin, bone turnover markers (BTMs), bone metabolism

## Abstract

Romosozumab is a monoclonal antibody against sclerostin that initially exhibits potent anabolic effects in treating osteoporosis. However, its efficacy diminishes after 6 mo, with bone formation markers declining despite continued therapy. We hypothesized that increased levels of Dickkopf-1 (Dkk1), a Wnt pathway inhibitor, may contribute to this attenuation by suppressing osteoblast activity. We conducted a 12-mo prospective observational study on postmenopausal osteoporosis naïve to anti-osteoporosis treatment treated with romosozumab. Serum levels of Dkk1, procollagen type I N-terminal propeptide (P1NP), C-terminal telopeptide of type I collagen (CTX), and sclerostin were measured at baseline (M0) and at 3 (M3), 6 (M6), and 12 mo (M12). BMD at the LS, FN, and TH was assessed at M0, M6, and M12. Associations between Dkk1 and P1NP were analyzed using linear mixed-effects models. Dkk1 levels increased significantly from 38.9 pmol/L at M0 to 44.2 pmol/L at M12 (*p* = .003). P1NP increased from 89.4 ng/mL at M0 to 115.4 ng/mL at M3 (*p* = .004) but decreased to 61.5 ng/mL by M12 (*p* < .001). CTX decreased significantly throughout the study (*p* < .001). BMD increased significantly at all sites by M12 (LS + 13.8%, FN + 6.3%, TH + 4.7%; all *p* < .01). An inverse association was found between Dkk1 increase and P1NP decrease between M3 and M12 (estimate = −0.909; *p* = .032). Romosozumab treatment is associated with a significant rise in Dkk1 levels, which correlates with a decrease in bone formation markers over time. Dkk1 may attenuate the anabolic effects of romosozumab by inhibiting Wnt signaling.

## Introduction

Osteoporosis is a chronic skeletal disorder characterized by decreased bone mass and increased fracture risk.^1^ Romosozumab, a monoclonal antibody against sclerostin, is a potent anabolic therapy for osteoporosis.^2^^,^^3^ By inhibiting sclerostin, romosozumab promotes osteoblast activity and bone formation while simultaneously decreasing bone resorption. Clinical trials have demonstrated significant increases in BMD and reductions in fracture risk with romosozumab treatment.^2^^,^^3^ The anabolic effects of romosozumab appear to diminish after 6 mo of continuous therapy. Markers of bone formation, such as procollagen type I N-terminal propeptide (P1NP), increase significantly during the first 3-6 mo of treatment but subsequently decline, even while treatment continues. Concurrently, bone resorption markers remain suppressed, indicating a shift from an anabolic to a predominantly anti-resorptive effect. The mechanisms underlying this attenuation of anabolic activity are not fully understood. Interestingly, individuals with sclerosteosis, a rare genetic disorder caused by loss-of-function mutations in the SOST gene encoding sclerostin, exhibit continuous bone formation without an early plateau.^4^ These patients experience progressive bone overgrowth, leading to hyperostosis and other skeletal abnormalities.^5^ The sustained absence of sclerostin results in uncontrolled osteoblast activity and bone accrual over many years. The discrepancy between the prolonged bone formation seen in sclerosteosis and the transient anabolic response to romosozumab raises critical questions. Specifically, why does pharmacological inhibition of sclerostin not replicate the continuous bone-building effects observed in genetic sclerostin deficiency? One plausible explanation involves compensatory upregulation of other Wnt pathway inhibitors, such as Dickkopf-1 (Dkk1). Dkk1 is a secreted protein that, like sclerostin, antagonizes Wnt/β-catenin signaling, thereby inhibiting osteoblast function and bone formation.^6^^,^^7^ In a previous study from our group, we showed that teriparatide was associated with a significant increase in Dkk1 serum levels, which was, in part, responsible for the waning of its anabolic effect.^8^ We hypothesize that prolonged inhibition of sclerostin by romosozumab leads to increased expression of Dkk1 as a compensatory feedback mechanism. Elevated levels of Dkk1 could suppress Wnt signaling despite ongoing sclerostin inhibition, resulting in decreased osteoblast activity and attenuation of the drug’s anabolic effects over time.

To investigate this hypothesis, we conducted a prospective study assessing the impact of romosozumab on serum Dkk1 and its association with bone turnover markers (BTMs) in postmenopausal women with severe osteoporosis.

## Materials and methods

We conducted a 12-mo prospective observational study on postmenopausal women with severe osteoporosis starting romosozumab 210 mg/monthly for 12 mo.

Inclusion criteria were:

- Treatment with romosozumab as deemed necessary by the treating physician.

Exclusion criteria were:

- History of myocardial infarction or stroke.
- Bone diseases other than osteoporosis (eg, Paget’s disease or osteomalacia).
- History of bone malignancy.
- Severe liver or kidney disease (eGFR <30 mL/min or Child–Pugh grade B/C).
- Uncontrolled endocrine disorders (eg, hypocalcemia, primary hyperparathyroidism).
- No exposure to bisphosphonates within 12 mo from romosozumab initiation.
- Naïve to any other anti-osteoporosis medications.

### Data collection

Patients were seen at baseline (M0, romosozumab initiation), after 3 (M3), 6 (M6), and 12 mo (M12, romosozumab stop). BMD tests were performed at M0, M6, and M12, at the FN, TH, and LS (L1-L4) using DXA with the QDR Hologic Delphi machine. The variation coefficient for the vertebral site was 1%, while it was 1.2% for the FN. Vertebral fracture assessment was performed. Blood samples were collected, fasting, in the morning at M0, M3, M6, and M12. The serum samples were aliquoted and stored at −80 °C until they were assayed in single batch. The analysis included the following biomarkers: C-terminal telopeptide of type I collagen (CTX, indicative of bone resorption), procollagen I intact N-terminal peptide (P1NP, a marker for bone formation), Dkk1 (a Wnt signaling inhibitor), and sclerostin (another Wnt inhibitor). CTX and P1NP were measured using the IDS-ISYS Multi-Discipline Automated Analyzer, utilizing chemiluminescence technology. The intra-assay variation was 3.0% for P1NP and 2.0% for CTX. Dkk1 and sclerostin levels were determined via ELISA kits with sensitivities of 0.89 and 8.9 pmol/L, respectively, and intra-assay variation coefficients of 7.8% for Dkk1 and 5.6% for sclerostin. Their inter-assay variations were 8.2% for Dkk1 and 6.9% for sclerostin.

To test the sclerostin assay accuracy and specificity, we recruited 4 healthy volunteers (3 females aged 28, 51, and 54 and 1 male aged 34). From each participant, we collected serum samples and divided them into 6 aliquots of 20 μL each. To simulate potential assay interference by romosozumab, we added increasing concentrations of romosozumab directly into these aliquots, as follows: 0 pmol/L (no romosozumab, control), 20 pmol/L (equivalent to the theoretical circulating concentration immediately after a single injection, calculated as detailed below), 100, 200, 400, and 800 pmol/L.

Concentrations were calculated based on the known amount of romosozumab per vial (105 mg/1.17 mL = 616 955 669 pmol/L). Assuming a worst-case scenario of uniform distribution of one entire vial of romosozumab into approximately 5 L of blood in an adult, this would result in approximately 123 391 134 pmol per liter of blood. Consequently, a 20 μL serum aliquot would contain approximately 20 pmol/L under these extreme conditions, and higher concentrations (100-800 pmol/L) represent multiples beyond physiological levels to robustly test for assay interference.

### Sample size considerations

The primary objective of the present study was to investigate the changes in Dkk1 serum levels in response to romosozumab treatment. The effect size derived from our previous study with teriparatide was 0.503 (Cohen’s δ − small to medium effect size).^8^ We conservatively hypothesized that Dkk1 would increase after romosozumab treatment with a similar, yet not higher, effect size. Therefore, we would need a sample size of 52 patients to reliably (with probability ≥0.8) detect an effect size of |δ| ≥ 0.4, assuming a two-sided criterion for detection that allows for a maximum Type I error rate of α = .05.

### Statistical analysis

Group comparisons were performed with ANOVA post-hoc tests. Categorical variables were compared with the χ^2^ test. All differences were considered significant when *p* value was inferior to .05. Absolute changes in bone markers were assessed with a mixed model for repeated measures (MMRM) using Satterthwaite and restricted maximum likelihood method with time, and baseline marker as fixed effects and with patients as random effect. Associations between continuous variables were tested using mixed linear models. A linear mixed-effects model was employed to analyze the relationship between bone formation marker P1NP and the Wnt inhibitor Dkk1 over time. The model included P1NP as the dependent variable and time (M3, M6, and M12) as well as Dkk1 as fixed effects. In our study, we focused on the attenuation (waning) phase of bone formation beyond M3-M12, since we hypothesized that the rise in serum Dkk1 levels would attenuate the anabolic response to romosozumab treatment only after the initial anabolic phase. To account for the potential interaction between the temporal changes in Dkk1 and their influence on P1NP, an interaction term (Dkk1^*^time) was included. Patient ID (ptID) was treated as a random effect to control for intra-subject variability across the repeated measures at different time points. Other covariates (eg, bone markers) were evaluated for inclusion based on biological relevance, correlations with the primary variables, and model fit criteria, such as Akaike information criterion and likelihood ratio tests. Markers with multicollinearity were excluded using variance inflation factor assessment. To account for multiplicity, we used the false discovery rate (FDR) approach with the two-stage step-up method of Benjamini, Krieger, and Yekutieli (*Q* value 5% of FDR). Missing data were imputed using a Random Forest algorithm Orange (Version 3.37.0). The imputation process was iterative and used a Random Forest model with 10 trees, the model allowed for an unlimited number of features and unrestricted tree depth. Splitting of nodes continued until each node contained fewer than five instances. All statistical analyses were performed using SPSS Version 26 (SPSS, Inc.), GraphPad Prism version 9.5.1 (GraphPad Software) and JASP (Version 0.19.0). This study was approved by the University of Verona ethic committee (prot. registration: REUMABANK). All patients provided informed consent to participate in the study.

## Results

**Figure 1:**
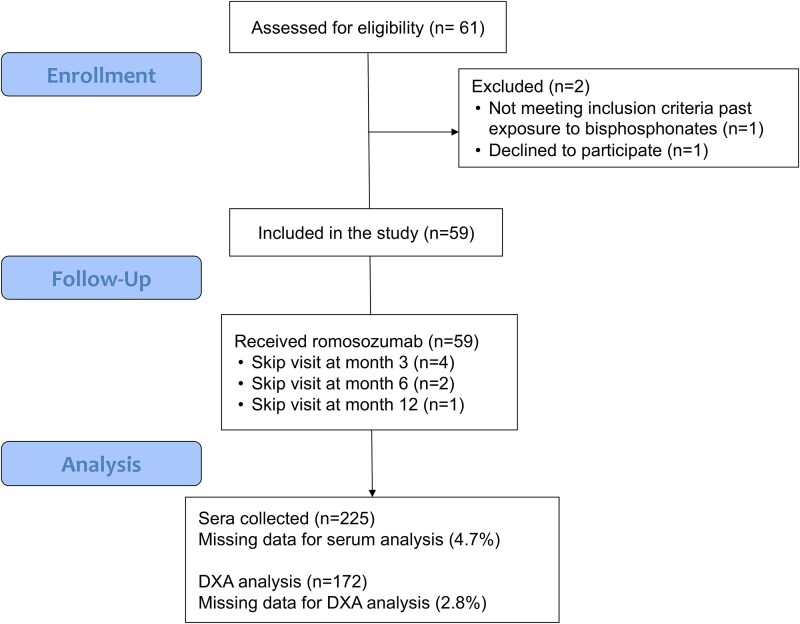
Flowchart of the study population.

Fifty-nine postmenopausal women were included in the study. Table 1 and Figure 1 show the baseline characteristics and disposition of study population.

**Table 1:** Baseline study population characteristics.

Characteristics	*n* = 59
**Age, years (SD)**	72.45 (9.54)
**BMI, kg/m^2^ (SD)**	23.1 (3.2)
**Age at menopause, years (SD)**	49.17 (4.37)
**Smoking status, *n* (%)** ** Former** ** Active** ** No smoker**	4 (6.8) 5 (8.5) 50 (85.7)
**Time since last fracture, months (IQR)**	28 (18-41)
**MOF: vertebral present (%)**	44 (74.6)
**Recent vertebral fracture—within 6 mo (%)**	2 (3.4)
**MOF: hip present (%)**	12 (20.3)
**Recent hip fracture—within 6 mo (%)**	1 (1.7)
**MOF: wrist present (%)**	5 (8.5)
**Recent wrist fracture—within 6 mo (%)**	1 (1.7)
**MOF: humerus present (%)**	3 (3.2)
**Recent humerus fracture—within 6 mo (%)**	0 (0)
**Non-MOF fractures, *n* (%)**	11 (18.6%)
**CTX, ng/mL (IQR)**	0.424 (0.348-0.555)
**P1nP, ng/mL (IQR)**	89.4 (56.5-108.5)
**25-OH-vitamin D, ng/mL (IQR)**	38.1 (28.6-46.3)
**PTH, pmol/L (IQR)**	4.4 (3.6–6.4)
**Sclerostin, pmol/L (IQR)**	34.0 (24.5-46.8)
**Dkk1, pmol/L (IQR)**	36.3 (24.9-48.6)
**Lumbar spine BMD, g/cm^2^ (SD)**	0.807 (0.156)
**Femoral neck BMD, g/cm^2^ (SD)**	0.646 (0.109)
**Total hip BMD, g/cm^2^ (SD)**	0.671 (0.117)
**Comorbidities** ** Hypertension (%)** ** Dyslipidemia (%)** ** Type 2 diabetes (%)** ** Peripheral Arterial Disease (%)** ** MGUS (%)** ** DVT (%)** ** VTE (%)** ** Hypothyroidism (%)** ** Atrial fibrillation (%)**	17 (28.8) 22 (37.3) 7 (11.9) 2 (3.4) 1 (1.7) 1 (1.7) 1 (1.7) 2 (3.4) 4 (6.8)
**Medications** ** Low dose aspirin (%)** ** Clopidogrel (%)** ** Direct-acting oral anticoagulants (%)** ** Statins (%)** ** ARBs and ACE inhibitors (%)** ** Calcium-antagonists (%)** ** Beta-blockers (%)** ** Proton pump inhibitors (%)** ** Thyroid hormone replacement (%)** ** SSRIs (%)** ** NSAIDs (intermittent use) (%)**	7 (11.9) 2 (3.4) 4 (6.8) 22 (37.3) 13 (22.0) 10 (16.9) 7 (11.8) 8 (13.5) 2 (3.4) 4 (6.8) 10 (16.9)

**Figure 2:**
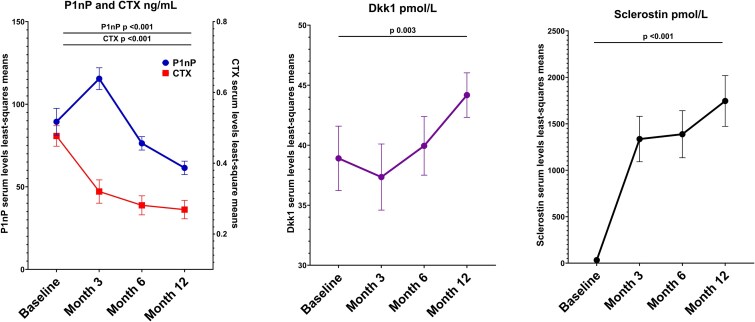
Bone turnover markers, Dkk1, and sclerostin serum levels during romosozumab treatment. Error bars represent standard error of the mean (SEM) for the least-square mean (LSM) derived from the mixed model for repeated measures.

Serum markers changes (least square changes from marginal means of the MMRM) are shown in Figure 2. Dkk1 increased significantly throughout the study period from 38.9 pmol/L at M0 to 44.2 pmol/L at M12 (*p* = .003, Cohen’s δ = 0.327). As expected P1NP increased between M0 and M3 (from 89.4 ng/mL SD 62.2 to 115.4 ng/mL SD 50.7, *p* = .004) and then slowly decreased through M12 (from 115.4 ng/mL SD 50.7 to 61.5 ng/mL SD 31.3, *p* < .001). CTX decreased significantly from M0 to M12 (from 0.477 ng/mL SD 0.221 to 0.269 ng/mL SD 0.196, *p* < .001). We found a steep increase in sclerostin serum levels as soon as M3 (from 36.3 pmol/L SD 15.6 to around 1500 pmol/L at all time points, *p* < .001).

**Figure 3:**
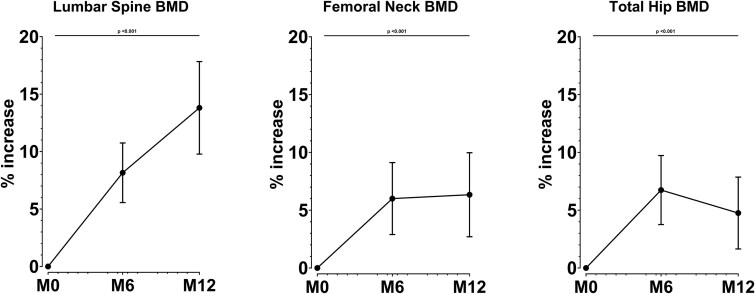
Changes in BMD in postmenopausal women treated with romosozumab*.* Error bars represent standard error of the mean (SEM) for the least-square mean (LSM) derived from the mixed model for repeated measures.

BMD changes are shown in Figure 3. Lumbar spine BMD increased significantly at M6 and M12 (+8.2% SD 9.9 *p* < .001 and +13.8% SD 15.4 *p* < .001, respectively). Femoral neck BMD increased significantly at M6 and M12 (+6.0% SD 11.9 *p* < .001 and +6.3% SD 13.9 *p* = .002, respectively). Total hip BMD increased significantly at M6 and M12 (+6.7% SD 11.4 *p* < .001 and +4.7% SD 11.9 *p* = .007, respectively).

**Figure 4:**
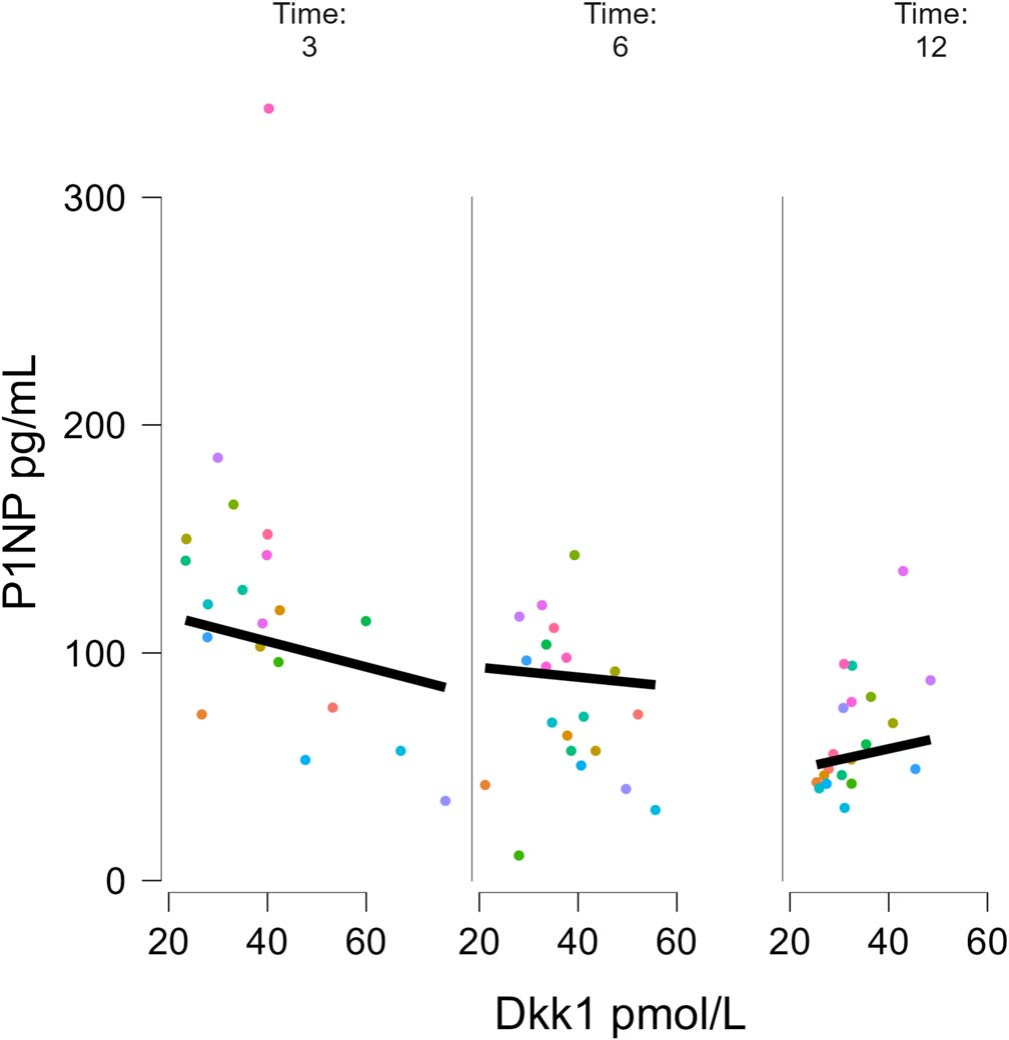
Association between P1NP serum levels and Dkk1 at different time points during romosozumab treatment.

In the linear mixed model Dkk1 increase between M3 and M12 was significantly associated with P1NP decrease between M3 and M12 (estimate −0.909 SE 0.419, *p* value = .032). This corresponds to a decrease in 1 ng/mL of P1NP every 0.909 pmol/L of Dkk1. We also found an interaction between Dkk1^*^time, albeit not significant (estimate 0.116 SE 0.067, *p* value = .089), this translates into an increase of the negative association between P1NP and Dkk1 every month beyond M3. Figure 4 show the association between P1NP and Dkk1 over the time beyond M3.

In our in-vitro experiment on the sera of healthy volunteers testing the sclerostin assay validity, we observed that measured serum sclerostin concentrations remained essentially unchanged across all aliquots, regardless of the increasing concentration of romosozumab added (Figure S1). The minor variations observed were within the range expected due to the inherent coefficient of variation (CV) of the ELISA assay itself.

## Discussion

In this 12-mo prospective observational study, we evaluated the effects of romosozumab on serum biomarkers and BMD in postmenopausal women with severe osteoporosis. Our results demonstrated, as expected, significant increases in BMD at the LS, FN, and TH regions. Parallel with the BMD improvements, we observed changes in BTMs. Serum P1NP, a marker of bone formation, increased significantly between baseline and M3 but declined thereafter, reaching levels lower than baseline by 12 mo. Conversely, CTX, a marker of bone resorption, decreased significantly throughout the study period.

A key observation was the significant increase in serum Dkk1 levels from baseline to 12 mo. Dkk1 is a known inhibitor of the Wnt signaling pathway, which is crucial for osteoblast function and bone formation.^6^^,^^7^ The negative association between rising Dkk1 levels and decreasing P1NP suggests that the upregulation of Dkk1 may counteract the anabolic effects of romosozumab over time. This feedback mechanism could contribute to the waning of bone formation despite continued therapy. Indeed, we found a significant association between decrease in P1NP levels between M3 and M12 with Dkk1 increases. Overall, the Dkk1 increase might explain the waning of the anabolic effects of romosozumab.

Our findings are consistent with previous studies indicating that romosozumab induces early increases in bone formation markers and BMD, followed by a plateau or decline in bone formation activity. The compensatory rise in Wnt pathway inhibitors like Dkk1 may partly explain this pattern. Yet, Dkk1 increase was moderate in magnitude (about 20%) and many other compensatory mechanisms are likely to explain the waning of the anabolic effect of romosozumab. Moreover, the negative association between serum Dkk1 and P1NP was particularly pronounced between months 3 and 6. Interestingly, the negative relationship diminished and became less evident (or even slightly positive) at month 12. A plausible biological interpretation for this observation is that the negative feedback exerted by increased Dkk1 levels on osteoblast activity is strongest during the earlier phase of treatment (between M3 and M6). Subsequently, at month 12, the natural pharmacological reduction of P1NP, an expected phenomenon during prolonged romosozumab treatment, reaches a plateau.

An interesting parallel can be drawn between our findings and those reported in our study investigating the long-term effects of teriparatide on Dkk1 in postmenopausal women with osteoporosis.^8^ Teriparatide, beyond 12 mo, induced a notable rise in serum Dkk1 levels, 26.9% at month 12% and 29.7% at month 18, coinciding with a decline in the pharmacological effect of the drug on bone formation markers. The consistent upregulation of Dkk1 observed in both studies suggests that elevated Dkk1 may serve as a common compensatory mechanism that limits the sustained anabolic effects of osteoporosis treatments targeting osteoblasts. Nonetheless, in contrast to what observed with teriparatide, with romosozumab we observed a suppression of resorption markers that started early after treatment initiation and lasted through month 12. We could therefore speculate that Dkk1 might exert a more potent inhibitor effect on osteoblasts, as opposed to sclerostin that has its prevalent function on osteoclasts. This is somehow in line with previous observations in multiple myeloma and rheumatoid arthritis, conditions characterized by high Dkk1 and prominent deficit of bone defect repair.^9^^,^^10^

A contrasting observation is seen with denosumab, an anti-resorptive agent targeting RANKL.^11^ In a 36-mo placebo-controlled trial, denosumab led to significant decreases in serum Dkk1 levels within the first 6 mo, with continuous reductions from the 18th month onward. The sustained decrease in Dkk1 during denosumab therapy could indeed explain the continuous increase in BMD observed over 10 years with denosumab, highlighting the potential role of increased Dkk1 levels in attenuating the anabolic response of romosozumab.

Interestingly, AGA2118 a novel agent that inhibits both sclerostin and Dkk1, showed promising increases in BMD in a phase 1 clinical trial and in animal studies.^12^ However, dual inhibition of Wnt antagonists might raise safety concerns. A previous dual inhibitor developed in 2016^13^^,^^14^ was probably withdrawn from development due to adverse effects, including increased skull thickness in monkeys leading to potential neurological issues.^15^ These findings highlight the necessity for cautious advancement of such therapies, ensuring that efficacy does not come at the expense of safety.

Our findings are contrasted by observations in patients with sclerosteosis and van Buchem disease. A study found significantly higher levels of serum Dkl1 levels in these patients compared to carriers and healthy controls.^16^ Despite the elevated Dkk1, the patients continued to exhibit hyperostotic phenotypes. This suggests that while a negative feedback mechanism via increased Dkk1 exists, it is insufficient to counteract the lack of sclerostin and the resultant overactivation of the Wnt signaling pathway in these genetic disorders. The discrepancy between the persistent bone overgrowth in sclerosteosis despite high Dkk1 levels and the attenuation of romosozumab’s anabolic effects raises questions about the underlying mechanisms. One possibility is that the pharmacological inhibition of sclerostin by romosozumab does not fully replicate the complete absence of sclerostin seen in sclerosteosis (ie, compensatory increase in the production of sclerostin). Additionally, the degree of Wnt pathway activation and the compensatory increase in Dkk1 might differ between the genetic absence of sclerostin and its pharmacological inhibition. In sclerosteosis, the chronic and absolute lack of sclerostin could lead to such a robust activation of Wnt signaling that even elevated Dkk1 levels cannot adequately suppress bone formation. Conversely, during romosozumab therapy, the upregulation of Dkk1 might be sufficient to dampen the drug’s anabolic effects over time. Interestingly, sclerosteosis patients, after puberty, reach a stable bone metabolism state without clear increases or changes in LS or FN BMD.

Regarding sclerostin levels, we observed a steep increase as early as 3 mo into treatment, with levels exceeding 1500 pmol/L at all subsequent time points. Such an exaggerated elevation is unlikely to represent a true physiological increase in active sclerostin. It was plausible that this finding is due to the interference of the ELISA assay with romosozumab itself. As an anti-sclerostin monoclonal antibody, romosozumab may form complexes with sclerostin that persist in circulation. The ELISA assay could be detecting these sclerostin-antibody complexes or cross-reacting with the therapeutic antibody, leading to artificially elevated sclerostin measurements. For this reason, we conducted the in-vitro experiment on sera of healthy volunteers. We hypothesized that the ELISA assay targets a specific, presumably inactive epitope of sclerostin, distinct from the active binding site of romosozumab (the region responsible for interaction with LRP5/6). Thus, even when sclerostin is bound by romosozumab at its active site, our assay still reliably measures the protein because it targets a different region. The steep increase in sclerostin levels observed in our patient samples treated with romosozumab (in vivo) can thus be plausibly explained by sequestration of sclerostin, originally bound in bone tissue, now detectable in serum in higher amounts bonded to romosozumab. Yet, between M3 and M12 we found a slight, yet not significant, further increase in sclerostin levels, this could reflect a compensatory biological response, but we cannot draw strong conclusions from our results. However, it is plausible that sclerostin truly increased in a sort of compensatory mechanism. In fact, the phase 1 study of romosozumab demonstrated that higher drug doses (10 mg/kg) led to a more pronounced stimulation of bone anabolism, supporting this hypothesis.^17^

Limitations of our study include the lack of a control group and the relatively small sample size, which may limit the generalizability of the results. Additionally, observational design precludes establishing causality.

In conclusion, romosozumab significantly improves BMD in postmenopausal women with severe osteoporosis but is associated with an increase in Dkk1 levels that may attenuate its anabolic effect over time. The development of therapies that can address this compensatory mechanism, such as dual inhibitors of sclerostin and Dkk1, holds potential but must be approached with careful consideration of safety profiles.

## Supplementary Material

RomoDkk1_supplementarymaterials_zjaf110

## Data Availability

Data of the analysis is available upon reasonable request.
