# Supplementary material for: Increase in serum DKK1 levels attenuates the anabolic response to romosozumab in postmenopausal osteoporosis
Source: J Bone Miner Res. 2025 Aug 19;41(2):143–9. doi: 10.1093/jbmr/zjaf110 (PMC12865849; doi:10.1093/jbmr/zjaf110)

**Supplementary materials**

**Increase in Serum DKK1 Levels Attenuates the Anabolic Response to Romosozumab in Postmenopausal Osteoporosis**

**Authors:** Giovanni Adami^1^, Filippo Montanari^1^, Angelo Fassio^1^, Francesco Pollastri^1^, Anna Piccinelli^1^, Camilla Benini^1^, Emma Pasetto^1^, Mattia Tugnolli^1^, Davide Gatti^1^, Maurizio Rossini^1^, Ombretta Viapiana^1^

1. Rheumatology Unit, University of Verona, Verona, Italy

**Table of Contents**

**Figure S1.** Sclerostin levels (pmol/L) in sera of healthy volunteers with increasing concentrations of romosozumab

**Figure S1.** Sclerostin levels (pmol/L) in sera of healthy volunteers with increasing concentrations of romosozumab


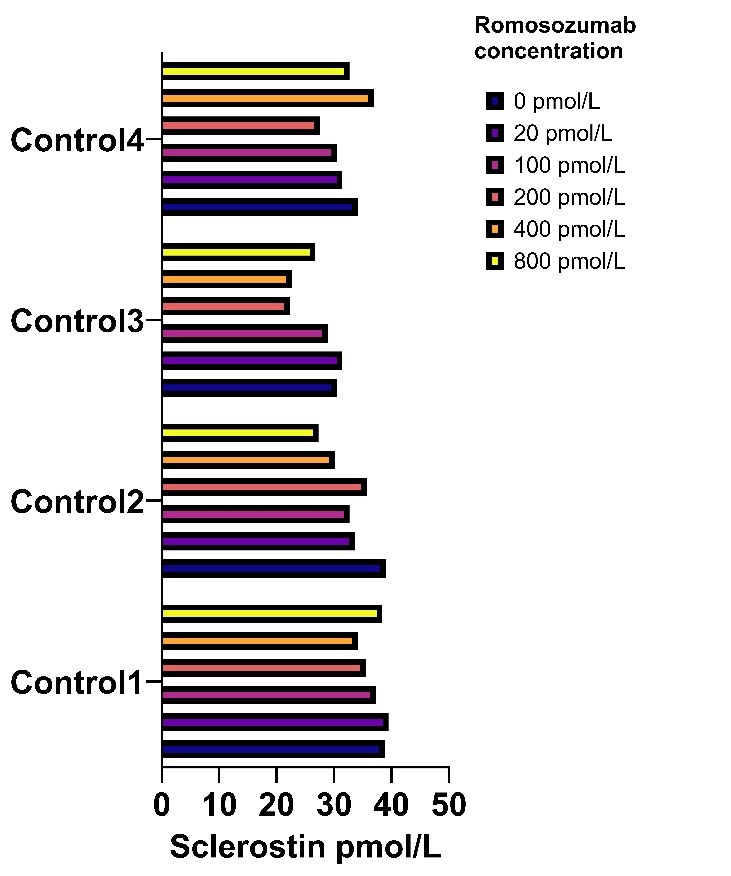

Supplement: RomoDkk1_supplementarymaterials_zjaf110 [file romodkk1_supplementarymaterials_zjaf110.docx]
